# DNA Damage Repair Pathway Alterations and Immune Landscape Differences in Pediatric/Adolescent, Young Adult (AYA) and Adult Sarcomas

**DOI:** 10.3390/cancers17121962

**Published:** 2025-06-12

**Authors:** Kurt Statz-Geary, Andrew Elliott, Steven Bialick, César Serrano, Margaret von Mehren, Matthew Oberley, Andrea P. Espejo-Freire, Priscila Barreto Coelho, Philippos A. Costa, Gina Z. D’Amato, Emily Jonczak, Jonathan C. Trent, Elizabeth Montgomery, David Lombard, Andrew Rosenberg, Aditi Dhir

**Affiliations:** 1 Miller School of Medicine/Sylvester Comprehensive Cancer Center, University of Miami, Miami, FL 33136, USA; k.statzgeary@med.miami.edu (K.S.-G.); steven.bialick@med.miami.edu (S.B.); priscila.barretocoe@jhsmiami.org (P.B.C.); gina.damato@med.miami.edu (G.Z.D.); emily.jonczak@med.miami.edu (E.J.); jtrent@med.miami.edu (J.C.T.); eam305@med.miami.edu (E.M.); dbl68@med.miami.edu (D.L.); arosenberg@med.miami.edu (A.R.); 2 Jackson Memorial Hospital, Miami, FL 33136, USA; 3 Caris Life Sciences, Phoenix, AZ 85040, USA; aelliott@carisls.com (A.E.); moberley@carisls.com (M.O.); 4 Vall d’Hebron Institute of Oncology, 08035 Barcelona, Spain; cserrano@vhio.net; 5 Fox Chase Cancer Center, Philadelphia, PA 19111, USA; margaret.vonmehren@fccc.edu; 6 Masonic Cancer Center, University of Minnesota, Minneapolis, MN 55455, USA; espej018@umn.edu; 7 Yale Cancer Center/Smilow Cancer Hospital, New Haven, CT 06519, USA; philippos.costa@yale.edu; 8 Department of Pathology and Laboratory Medicine, and Sylvester Comprehensive Cancer Center, Miller School of Medicine, Miami VA Healthcare System, Miami, FL 33136, USA

**Keywords:** sarcoma, precision medicine, DNA damage response

## Abstract

**Background:** DNA damage response (DDR) pathway alterations contribute to genomic instability and malignant progression in several cancers. **Methods**: We retrospectively reviewed molecular profiles from 5309 sarcoma patient samples, including 746 from pediatric/adolescent and young adults (ped/AYA), encompassing 38 histologic subtypes. The gene expression profiles were further analyzed for immunotherapy-related biomarker associations, including analysis of the T cell-inflamed score. **Results**: Pathogenic/likely pathogenic DDR alterations were detected in 15.9% (*N* = 842) of samples overall and 9.25% (*N* = 69) of Ped/AYA tumors, with mutations occurring most frequently in *ATRX* (10.1%). Shorter overall survival was observed for patients with DDR-alterations compared to those with DDR-wildtype tumors (Hazard Ratio = 1.172, 95% CI: 1.068–1.287; *p* < 0.001). In many subtypes, DDR-mutated tumors were found to have increased rates of immune markers, including PD-L1+, dMMR/MSI-high, and TMB. **Conclusions:** Our study of somatic DDR-pathway mutations provides a better understanding of the molecular associations across sarcoma subtypes that may aid in developing future prognostic and therapeutic options for these rare cancers.

## Simple Summary

This article investigates the genetic and immunologic landscape of sarcomas with a focus on DNA Damage Response pathway alterations, stratified by specific histologic subtypes. To our knowledge, no other study to date has described this population of sarcomas with this level of precision. This research may aid in identifying precision treatment targets in sarcomas and stratifying patient outcomes by molecular markers.

## 1. Introduction

Sarcomas are rare malignancies of mesenchymal differentiation that comprise approximately 20% of pediatric and 1% of all adult cancers [1]. There are approximately 175 unique histologic subtypes, and their broad spectrum of morphological and biological features makes their accurate diagnosis and appropriate treatment challenging [1]. Adding to the challenges in treatment, up to 50% of soft tissue sarcomas will metastasize, and first-line therapy with anthracycline-based agents has a median progression-free survival of only 6 months [2]. Second-line therapy is often then selected based on molecular markers. Together, these challenges highlight the importance of better understanding the genetic landscape and possible targets for each histologic subtype [3]. Many sarcomas have pathogenic mutations in known and novel cancer genes, yet little is known about the role of DNA damage response (DDR) pathway alterations in their pathogenesis.

The DDR pathway plays an integral role in the repair of DNA mutations induced by a variety of environmental factors, such as ultraviolet light, smoking, and cancer treatments, and contributes to the prevention of tumor development [4,5]. This damage response occurs through several different pathways, including nucleotide excision repair (NER), base excision repair (BER), non-homologous end joining (NHEJ), alternative end joining, homologous recombination repair (HRR), single strand break repair (SSBR), and Fanconi anemia pathways (FA). DDR pathway gene alterations are associated with increased genomic instability and have been implicated in a variety of different carcinomas, including breast, ovarian, colon, urothelial, and numerous other cancers [6].

Recent studies of somatic alterations in bone and soft tissue sarcomas have noted an overall DDR alteration rate of 9.6%, with higher rates (>10%) noted in uterine leiomyosarcoma, malignant peripheral nerve sheath tumor, perivascular epithelioid cell tumor, angiosarcoma, pleomorphic liposarcoma, and osteosarcoma. [7,8,9,10]. In a large multi-platform profiling of over 2000 sarcoma specimens, *TP53* (26.3%) and *BRCA2* (17.6%) were identified as the most commonly mutated genes [11]. Investigations have provided evidence that DDR gene alterations may play an important role in sarcoma development [1,6,12]. A large international genetic study of patients with sarcoma identified pathogenic germline variants in known and novel cancer genes, including *TP53*, *ATM*, *ATR*, *BRCA2,* and *ERCC2* [6]. A recent meta-analysis also suggested that germline alterations of genes involved in DNA repair machinery could indicate a predisposition to sarcoma [1]. Chan et al. found that germline mutations in the DDR pathway had a prevalence of 10.6% in an Asian cohort of sarcoma patients [12]. In addition to the mutational landscape, immune signatures and tumor antigenicity findings also play a role in response to immunotherapy [13,14].

The role of mutations in the DDR pathway and immune landscape in pediatric, adolescent, and young adult (ped/AYA) patients with sarcoma is not well established, as most studies encompass all age groups or only adults. Due to molecular differences between pediatric/AYA and adult cancers, studies that do not stratify by age may have limited validity for pediatric patients. Age-related differences may partly result from accumulated somatic mutations over time, with an estimated increase of 0.077 mutations per megabase per year [15,16]. Accordingly, increased DNA damage repair signatures are observed in older patients [15,16]. Studies have shown age-related differences in the molecular profiles of soft tissue and osteosarcomas, offering potential for tailored therapies in pediatric and AYA patients [17,18]. For instance, one osteosarcoma study found that the mutations that predict sensitivity to multi-TKI treatment are more common in younger patients, while mutations predictive of sensitivity to CDK4/6 inhibitors predominated in adults, illustrating how understanding age-specific molecular trends can identify therapeutic targets [18]. Accordingly, we performed an in-depth analysis of somatic DDR pathway alterations across all sarcoma subtypes, as well as their association with immune signature in adults and ped/AYA subpopulation, to better understand the associated molecular features and their impact on patient outcomes.

## 2. Materials and Methods

### 2.1. Study Design

Retrospectively, we reviewed the molecular profiles of patients’ sarcoma specimens (total *N* = 5309, AYA/Pediatrics *N* = 746), representing 38 histologic subtypes. We classified and abbreviated the histological subtypes according to the OncoTree classification. This information is submitted as part of routine clinical care to a CLIA-certified laboratory (Caris Life Sciences^®^; Phoenix, AZ, USA) [19]. The pathological diagnoses accompanying the submitted tissues were used for the study. The study was conducted in accordance with the guidelines of the Declaration of Helsinki, the Belmont Report, and the U.S. Common Rule. In compliance with policy 45 CFR 46.101(b), this study was conducted using retrospective, de-identified clinical data, and patient consent was not required.

### 2.2. DNA Next-Generation Sequencing (NGS)

Genomic tumor DNA isolated from formalin-fixed paraffin-embedded (FFPE) tumor samples (*N* = 5194) was micro-dissected to enrich tumor purity and subjected to NGS using the NextSeq or NovaSeq 6000 platforms (Illumina, Inc., San Diego, CA, USA). For NextSeq sequenced tumors (*N* = 3121), a custom-designed SureSelect XT assay was used to enrich 592 whole-gene targets (Agilent Technologies, Santa Clara, CA, USA). For NovaSeq sequenced tumors (*N* = 2073), whole exome sequencing (WES) was performed, with more than 700 clinically relevant genes sequenced at high coverage and high read-depth, along with another panel designed to enrich for additional >20,000 genes at a lower depth. All variants were detected with >99% confidence based on allele frequency and amplicon coverage, with an average sequencing depth of coverage of >500 and an analytic sensitivity of 5%. Genetic variants identified were interpreted by board-certified molecular geneticists and categorized as ‘pathogenic’, ‘likely pathogenic’, ‘variant of unknown significance’, ‘likely benign’, or ‘benign’, according to the American College of Medical Genetics and Genomics (ACMG) standards. When assessing mutation frequencies of individual genes, the number of samples harboring ’pathogenic’ and ‘likely pathogenic’ variants was counted as mutated and divided by the total number of samples tested.

### 2.3. Gene Fusion Detection by Whole Transcriptome Sequencing

Gene fusion detection was performed on mRNA isolated from an FFPE tumor sample (*N* = 3612) using the Illumina NovaSeq platform (Illumina, Inc., San Diego, CA, USA) and Agilent SureSelect Human All Exon V7 bait panel (Agilent Technologies, Santa Clara, CA, USA). The FFPE specimens underwent pathological evaluation to determine the percent tumor content and tumor size; a minimum of 10% of tumor content in the area for microdissection was required to enable enrichment and extraction of tumor-specific RNA. The Qiagen RNA FFPE tissue extraction kit (Hilden, Germany) was used for extraction, and the RNA quality and quantity were determined using the Agilent TapeStation. Biotinylated RNA baits were hybridized to the synthesized and purified cDNA targets, and the bait-target complexes were amplified in a post-capture PCR. The resultant libraries were quantified, normalized and the pooled libraries were denatured, diluted, and sequenced; the reference genome used was GRCh37/hg19 and analytical validation of this test demonstrated ≥97% Positive Percent Agreement (PPA), ≥99% Negative Percent Agreement (NPA) and ≥99% Overall Percent Agreement (OPA) with a validated comparator method.

### 2.4. Genomic Scar Score (GSS) Calculation and Homologous Recombination Deficiency (HRD)

For samples profiled by WES, GSS was calculated as a composite of genomic loss of heterozygosity (gLOH) and large-scale transitions (*N* = 2138) [20]. For genomic gLOH calculation, 22 autosomal chromosomes were split into 552 segments, and the LOH of SNPs within each segment was calculated. Around 99% of segments were at least 5Mb in length; segments excluded from the calculation included those spanning ≥90% of a whole chromosome or chromosome arm and segments. Samples with a GSS ≥42 were considered HRD [20].

### 2.5. Real-World Cohort Survival Analysis

Overall survival was calculated using a repository of real-world evidence (RWE) insurance claim data, with overall survival defined from the biopsy date until the last contact. Patients that did not have an observed claim data element within 100 days of the end of available RWE records were presumed to be deceased and uncensored, which was found to be 95% concordant with data obtained from National Death Index data (National Center for Health Statistics, Centers for Disease Control and Prevention). All other patients were censored. Hazard ratios (HR) for survival (and 95% confidence intervals) were computed using the Cox proportional hazards model, HRs were calculated for the univariate model based on DDR mutation status and for a multivariate model that includes patient age and sex variables by sarcoma subtype. Overall survival between groups was compared using the log-rank test.

### 2.6. Statistical Analysis

Molecular associations were tested by Chi-square and Fisher’s exact tests for categorical variables and Mann–Whitney U for continuous variables, where appropriate. Statistical analyses were performed with JMP V13.2.1 (SAS Institute) and open-source Python (v3.9.7) libraries (Pandas, NumPy, Seaborn, and Matplotlib).

## 3. Results

### 3.1. Study Cohort Description

**Figure 1:**
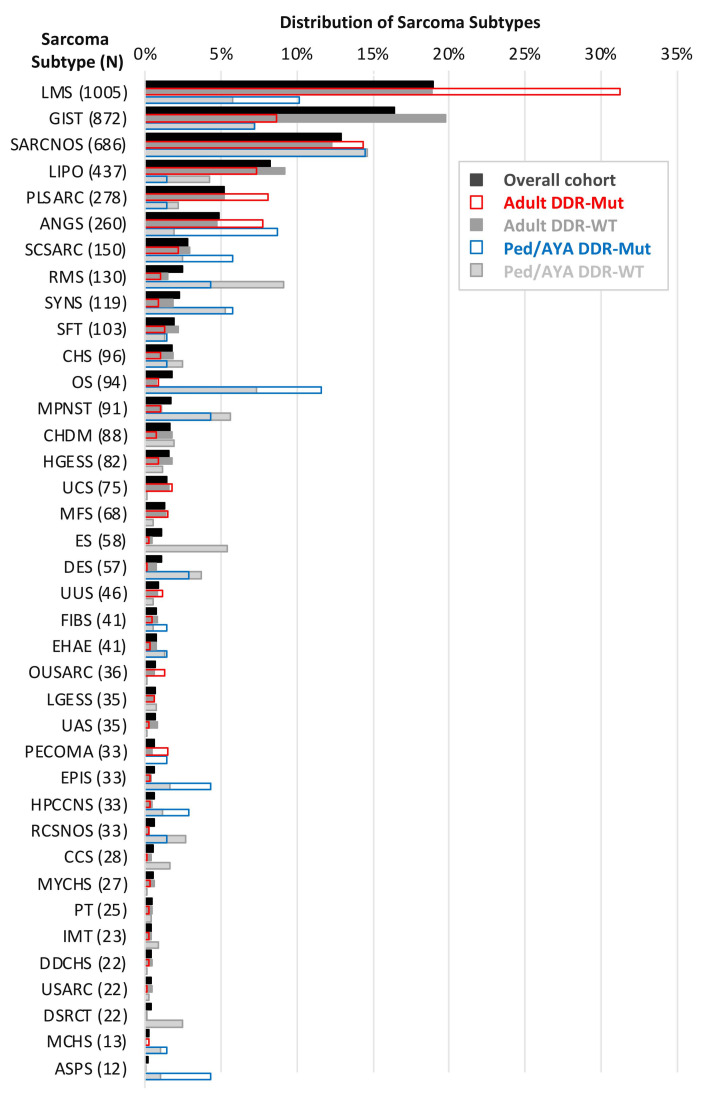
Distribution of sarcoma subtypes. Subtypes sorted by the proportion of the overall study cohort. Samples further stratified into DDR-mutant (Mut) and wildtype (WT) subgroups for adult and ped/AYA patients.

In total, there were 5309 sarcoma samples, representing 38 subtypes based on the OncoTree classification, with an overall median age of 60 years (range 0–90+ years) and 56.6% female patients (Table 1). The most common subtypes included leiomyosarcoma (*N* = 1005), gastrointestinal stromal tumors (*N* = 872), and liposarcoma (*N*= 437), as well as many samples reported as ‘sarcoma, not otherwise specified (NOS)’ (*N*= 686) (Figure 1). The total number of sarcoma subtypes associated with patient demographics and the proportion of ped/AYA patients by subtype are shown in Table 1. Adult DDR pathway-mutant (DDR-mut) samples were enriched in leiomyosarcoma, angiosarcoma, and pleomorphic sarcoma compared to wildtype (WT) (Figure 1). Among ped/AYA patients, leiomyosarcoma, angiosarcoma, osteosarcoma, and alveolar soft parts sarcoma were enriched in DDR-mut, while rhabdomyosarcoma and Ewing sarcoma were enriched in WT (Figure 1).

### 3.2. Landscape of DDR Pathway Alterations by Sarcoma Subtype

**Figure 2:**
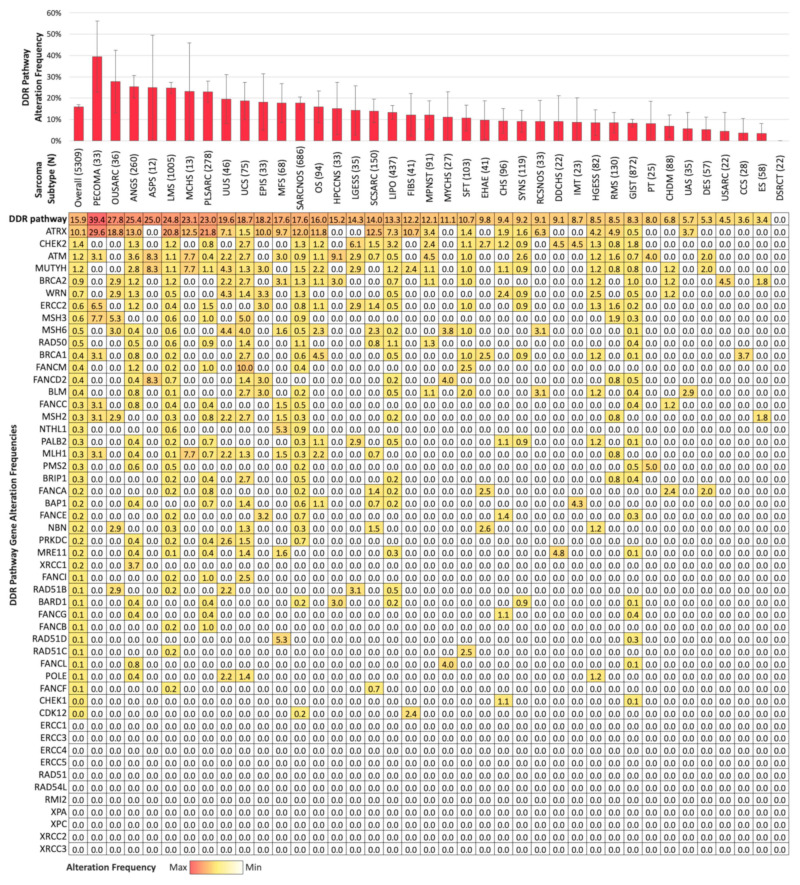
DDR pathway alteration landscape by sarcoma subtype. Bar graph represents overall DDR pathway alteration frequency by sarcoma subtype in descending order (error bars reflect 95% confidence interval). Table of alteration frequencies for each DDR pathway gene by sarcoma subtype, with rows sorted by descending overall alteration frequency.

Overall, pathogenic/likely pathogenic DDR pathway alterations were detected in 842 (15.9%) sarcoma samples (Figure 2). *ATRX* was the most frequently altered DDR gene (10.1% overall), with mutations observed across 24 sarcoma subtypes (11 subtypes with >10% mutation rate including perivascular epithelioid cell tumor, uterine sarcoma other, angiosarcoma, leiomyosarcoma, mesenchymal chondrosarcoma, pleomorphic sarcoma, epithelioid sarcoma, sarcoma NOS, osteosarcoma, spindle cell sarcoma, and fibrosarcoma. The next most frequently mutated genes included *CHEK2* (1.4%), *ATM* (1.2%), and *MUTYH* (1.2%), with the remaining DDR pathway genes altered in less than 1% of all sarcomas. Distinct patterns of DDR gene alteration frequencies were observed across histologic subtypes, with >20 subtypes identified with individual DDR genes altered in ≥3% of samples, including 3.0% of epithelioid sarcoma and 6.5% of perivascular epithelioid cell tumor samples harboring *ERCC2* mutations.

### 3.3. HRD Frequency and Association with DDR Alterations in Sarcomas

**Figure 3:**
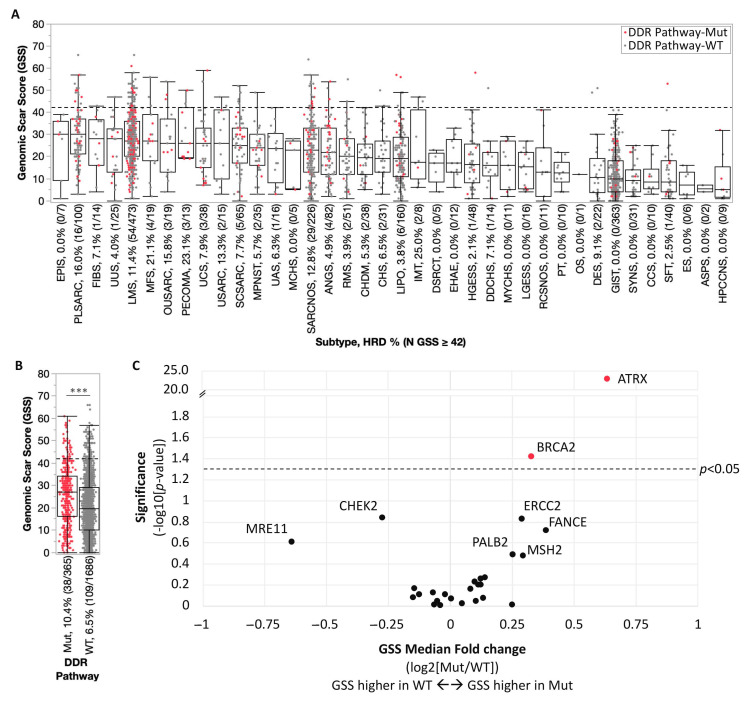
Association between DDR pathway alteration and HRD phenotype. (**A**) Genomic scar score (GSS) distribution by sarcoma subtype, with DDR pathway-mutated samples indicated in red. (**B**) Samples stratified by DDR pathway alteration status. Dashed line in A and B indicates threshold for presumed homologous recombination deficiency (HRD; GSS ≥ 42). (**C**) Volcano plot of GSS median fold changes for samples stratified by individual DDR gene alteration status. *** *p* < 0.001.

For samples profiled by WES, we examined sarcoma genomes for an HRD phenotype, often associated with DDR pathway alterations in various cancer types, by calculating a GSS that is predictive of poly adenosine diphosphate-ribose polymerase (PARP) inhibitor sensitivity [20]. The median GSS ranged from 5 to 30 across sarcoma subtypes, with the highest median values observed in epithelioid sarcoma and pleomorphic sarcoma, and the lowest median values observed in hemangiopericytoma of the central nervous system (Figure 3A). The highest rates of HRD (GSS ≥ 42) were observed in inflammatory myofibroblastic tumors (25%), perivascular epithelioid cell tumors (23.1%), myxofibrosarcoma (21.1%), pleomorphic sarcoma (16.0%), and other uterine sarcoma (15.8%), while low rates of HRD were observed in many subtypes, including 15 subtypes with no HRD samples identified. Median GSS was significantly increased in DDR pathway-altered sarcomas (27 vs. 19.5 in WT, *p* < 0.001), along with increased frequency of HRD (GSS ≥ 42; 10.4% vs. 6.5% in WT, *p* < 0.01) (Figure 3B). *ATRX* (1.6-fold, *p* < 0.0001) and *BRCA2* (1.3-fold, *p* < 0.05) were associated with significantly higher GSS compared to respective WT subgroups, and *FANCE* (1.3-fold, *p* = 0.19), *ERCC2* (1.2-fold, *p* = 0.15), *PALB2* (1.2-fold, *p* = 0.32), and *MSH2* (1.2-fold, *p* = 0.33) mutations showed a trend toward higher GSS, while a trend for lower GSS was observed for *MRE11* (0.64-fold, *p* = 0.24) and *CHEK2* (0.83-fold, *p* = 0.14) (Figure 3C).

### 3.4. DDR Alterations and HRD in Pediatric/AYA Sarcomas

**Figure 4:**
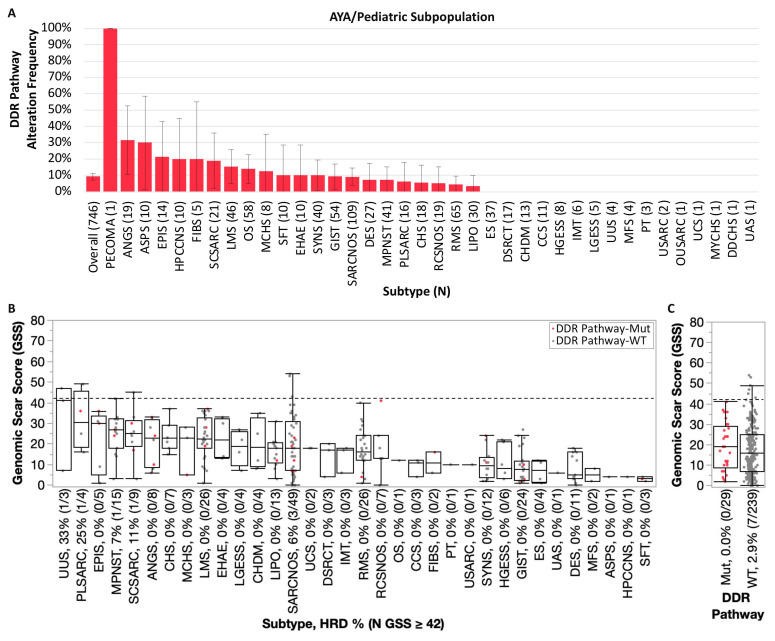
Pediatric/AYA DDR pathway alteration landscape and association with HRD phenotype. (**A**) Bar graph represents overall DDR pathway alteration frequency by sarcoma subtype in descending order (error bars reflect 95% confidence interval). (**B**) Genomic scar score (GSS) distribution by sarcoma subtype, with DDR pathway-mutated samples indicated in red. (**C**) Samples stratified by DDR pathway alteration status. Dashed line indicates threshold for presumed homologous recombination deficiency (HRD; GSS ≥ 42).

Within the ped/AYA patients (0–39 years at time of biopsy), the overall DDR pathway alteration rate was 9.25% (*N* = 69). Excluding perivascular epithelioid cell tumor due to limited sample size (*N* = 1), DDR pathway alteration rates were highest among angiosarcoma (6/19, 31.6%) and alveolar soft parts sarcoma (3/10, 30.0%) (Figure 4A). DDR pathway alteration frequencies ≥10% were also observed for epithelioid sarcoma, hemangiopericytoma of the central nervous system, fibrosarcoma, spindle cell sarcoma, leiomyosarcoma, osteosarcoma, mesenchymal chondrosarcoma, solitary fibrous tumor/hemangiopericytoma, epithelioid hemangioendothelioma, and synovial sarcoma. Consistent with the overall cohort, the most frequently mutated DDR pathway gene was *ATRX* (20/446, 4.3%), which was observed in 9 sarcoma subtypes (2.4–15.8% *ATRX*-mut). Similarly, other DDR gene mutations were observed in <1.5% overall.

Although ped/AYA samples profiled by WES were limited, median GSS ranged from 3 to 41 across sarcoma subtypes, with the lowest and highest medians observed in solitary fibrous tumor and undifferentiated uterine sarcoma, respectively (Figure 4B). A low HRD frequency was observed among ped/AYA sarcomas overall (2.6% vs. 7.9% in adult sarcomas, *p* = 0.002), with HRD samples limited to undifferentiated uterine sarcoma (33.3%, 1/3), pleomorphic sarcoma (25%, 1/4), spindle cell sarcoma (11.1%, 1/9), MPNST (6.7%, 1/15), and sarcoma NOS (6.1%, 3/49). However, ped/AYA DDR pathway-altered sarcomas were not associated with significantly higher GSS (median GSS 19 vs. 16 in WT, *p* = 0.36), and none were considered HRD, while HRD was observed in 2.9% (7/239) of DDR pathway-WT samples (Figure 4C).

### 3.5. Tumor Microenvironments and Immunotherapy-Related Biomarkers Associated with DDR Pathway Alterations

**Figure 5:**
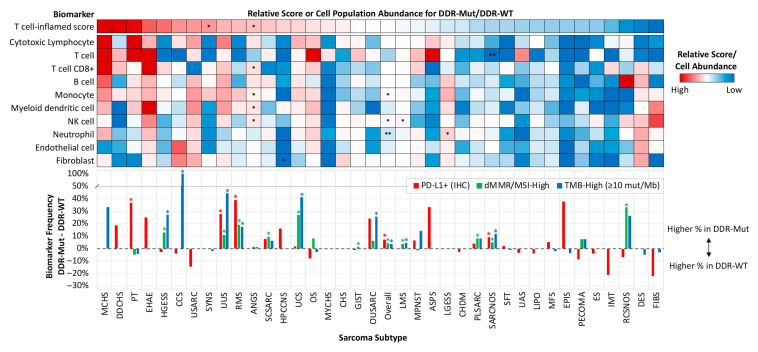
Landscape of tumor microenvironment features and immunotherapy biomarkers associated with DDR pathway mutation. Heatmap of the relative T cell-inflamed score, immune and stromal cell population abundance (MCP-counter), and common immunotherapy-associated biomarkers (PD-L1 IHC, dMMR/MSI-High, and TMB-High). Bar chart reflects the difference in biomarker frequency between DDR-mut and DDR-wt subgroups. * *p* < 0.05, ** *p* < 0.01.

The gene expression profiles of patient samples that underwent whole transcriptome sequencing were further analyzed for immunotherapy-related biomarker associations with DDR pathway alteration, including analysis of a gene expression signature that predicts for response to pembrolizumab (T cell-inflamed score) and quantification of the relative abundance of immune and stromal cell populations (MCP-counter) [21,22]. T cell-inflamed scores were significantly increased in DDR pathway-altered synovial sarcoma (+0.15 arbitrary units, AU) and angiosarcoma (+0.10 AU), with numerically increased median scores observed for several other subtypes, which correlated with increased abundance of pro-inflammatory cell populations in subtypes with higher T cell-inflamed scores (Figure 5). DDR pathway alterations were associated with increased frequency of PD-L1+ IHC overall (+7.3%) and among rhabdomyosarcoma (+39.1%), phyllodes tumor of breast (+37%), undifferentiated uterine sarcoma (+27.9%), and sarcoma NOS (+9.3%) subtypes (all *p* < 0.05). The frequency of dMMR/MSI-High and TMB-High was also increased in DDR pathway-altered sarcoma compared to DDR pathway-WT overall (+4.4% and +3.7%, respectively), as well as in high-grade endometrial stromal sarcoma; +13.0% and +27.2%), undifferentiated uterine sarcoma (+11.1% and +44.4%), rhabdomyosarcoma (+19.1% and +17.3%), uterine carcinosarcoma/uterine malignant mixed mullerian tumor; +26.9% and +41.2%), leiomyosarcoma (+3.8% and +4.6%), and sarcoma NOS(+4.9% and +11.9%) subtypes (all *p* < 0.05). Additional subtypes with an increased frequency of dMMR/MSI-High among DDR pathway-altered tumors included round cell sarcoma, NOS (+33.3%), spindle cell sarcoma (+9.5%), osteosarcoma (+8.3%), pleomorphic sarcoma (+8.3%), and gastrointestinal stromal tumor (+1.5%), while TMB-high frequencies were increased in DDR pathway-altered tumors among clear cell sarcoma (+95.8%) and other uterine sarcoma (+25.8% TMB-High) subtypes (all *p* < 0.05).

### 3.6. Clinical Outcomes Associated with DDR Pathway Alterations

**Figure 6:**
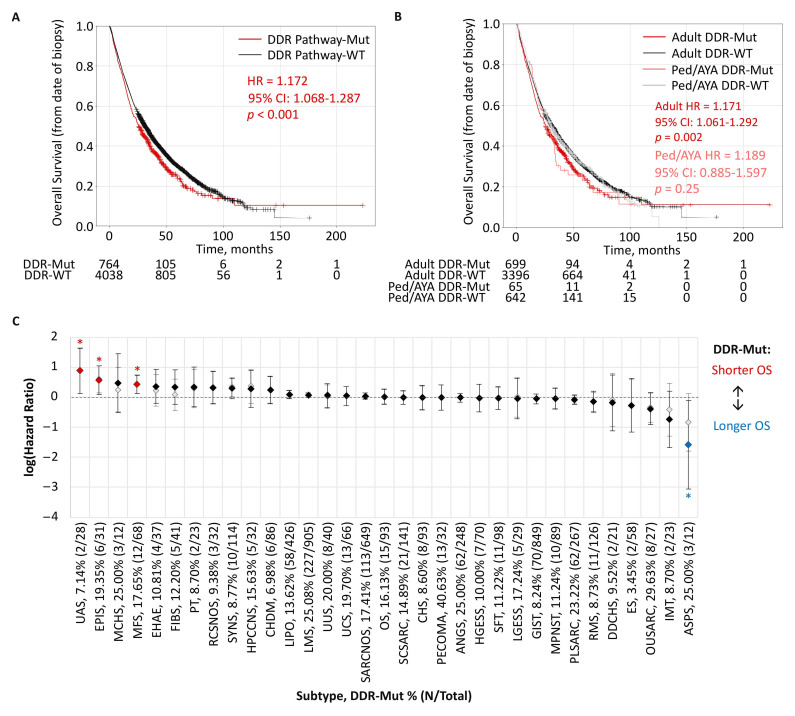
Clinical outcomes associated with DDR pathway alterations. (**A**) Kaplan–Meier plots of overall survival (OS) calculated from the date of biopsy for DDR pathway-mutated (Mut) and wildtype (WT) subgroups across the overall study cohort. (**B**) Kaplan–Meier plots of OS among adult and ped/AYA subpopulations. (**C**) Forest plot of OS for DDR pathway-Mut and WT subgroups across sarcoma subtypes in a univariate (gray diamonds, gray confidence intervals) and multivariate Cox proportional hazards model including age and sex variables (black/red/blue diamonds, black confidence intervals). * *p* <0.05.

To determine the potential clinical impact of DDR pathway alterations, real-world patient overall survival was determined from insurance claim-based data. Among 4802 patients with data available, patients harboring DDR pathway mutations (15.9%, N = 764) had significantly shorter overall survival compared to WT patients (HR = 1.172, 95% CI: 1.068–1.287, *p* < 0.001, Figure 6A). Further stratification by age groups showed DDR-mut was associated with shorter overall survival among both adult and ped/AYA patients, although not statistically significant among ped/AYA patients, in part due to the limited sample size of that subpopulation (Figure 6B). When stratifying by sarcoma subtype, DDR pathway mutations were associated with significantly shorter overall survival among uterine adenosarcoma, epithelioid sarcoma, and myxofibrosarcoma, while DDR mutations were associated with longer overall survival among patients with alveolar soft part sarcoma when adjusted for age and sex in a multivariate analysis (Figure 6C).

## 4. Discussion

Our analysis revealed an overall DDR pathway alteration rate among all histologic types of 15.86% (n = 842), compared to the 9.6% overall mutation rate reported in Nacev et al. [7], with the highest alteration rates observed for perivascular epithelioid cell tumor, other uterine sarcoma, angiosarcoma, alveolar soft parts sarcoma, and leiomyosarcoma (range 24.8–39.4%), while the lowest rates occurred in desmoplastic small-round-cell tumor, Ewing sarcoma, clear cell sarcoma, uterine sarcoma/mesenchymal, desmoid aggressive fibromatosis, and uterine adenosarcoma (range 0.00–5.71%). The higher rate of DDR mutations seen in our study may suggest that DDR mutations are more prevalent in sarcoma subtypes than previously thought. These data provide novel insight into the potential somatic DDR pathway alterations that can be encountered in several previously overlooked histologic sarcoma subtypes, such as perivascular epithelioid cell tumors and alveolar soft parts sarcoma. Additionally, the results of this study are consistent with previous observations of high rates of DDR pathway gene alterations in patients with leiomyosarcoma [9,14] and angiosarcoma [8], emphasizing the importance of considering DDR pathway alterations in these cancer subtypes.

A prior study of somatic DDR gene alterations in leiomyosarcoma found a 20% alteration rate, with the majority of these in the homologous recombination pathway. The authors also noted that non-*BRCA* homologous recombination alterations conferred a worse prognosis than *BRCA*-mutated and WT tumors [9]. Our study noted a similar DDR alteration rate in leiomyosarcoma (24%), with the majority of alterations in *ATRX*, which plays a role in HRR [23]. A separate study found that *BRCA2* mutations were present in 17% of leiomyosarcoma [11]. Prior studies of angiosarcoma noted high rates of *ATRX* mutation, high TMB, and *PDL-1* positivity, particularly among head and neck angiosarcoma [8], while we observed a DDR alteration rate of 25% and *ATRX* alteration rate of 13% among angiosarcoma.

Amongst pediatric/AYA patients, we noted the highest rates of DDR mutations among angiosarcoma, alveolar soft parts sarcoma, epithelioid sarcoma, hemangiopericytoma of the central nervous system, spindle cell sarcoma, leiomyosarcoma, osteosarcoma, mesenchymal chondrosarcoma, solitary fibrous tumor, epithelioid hemangioendothelioma, and synovial sarcoma, all with alteration rates of 10% or greater. The lowest rates of DDR alterations (excluding subtypes with *N* < 5) were among Ewing sarcoma, desmoplastic small-round-cell tumor, chordoma, clear cell sarcoma, inflammatory myofibroblastic tumor, and low-grade endometrial stromal sarcoma, all with 0% alteration rates. Germline pathogenic variants in DDR genes, including *FANCC*, *CHEK2*, and *FANCA*, have been reported as predisposing to the development of Ewing sarcoma [24]. A recent study investigated the molecular profiling of ped/AYA sarcomas and noted alterations in several DDR genes, including *SLFN11*, *EWSR1*, *BRCA1*, *BRCA2,* and *MLH1* [25]. However, the study had a relatively small number of patients identified with DDR alterations and did not investigate histology-specific alteration frequencies.

Sarcoma patients with germline pathogenic variants in genes associated with cancer risk had an earlier age of onset than those without, suggesting that these mutations play a role in tumorigenesis. Many of the genes implicated in this study were involved in DNA damage response, including *ATM*, *ATR*, *BRCA*, *TP53*, and *ERCC2*, providing further evidence that DDR genes play a role in sarcoma development [6]. Another study of germline mutations found DDR alterations present in 10.6% of sarcoma patients [12]. A limitation of our study is a lack of germline testing, and further evaluation is needed to determine the prevalence and implications of the hereditary alterations in sarcomas.

In addition to reporting overall somatic DDR pathway alteration rates, the mutation frequencies of individual DDR genes were studied to isolate specific genes that may be of pathogenic significance, as detailed below. The *ATRX* gene, a tumor suppressor and chromatin remodeler, demonstrated the highest mutation frequency (10.1%), particularly among leiomyosarcoma, consistent with other reports [10,23,26]. As recently noted in the literature, *ATRX* mutation was associated with a more aggressive sarcoma and worse overall survival compared to ATRX-wt [27]. Recent studies have found that *ATRX* alterations contribute to immune escape and increased tumor growth in pleomorphic sarcoma and that *ATRX*-deficient tumor cells were particularly susceptible to the WEE1 inhibitor AZD1775 [28,29]. Further research into targeting *ATRX* should consider multiple sarcomas, particularly leiomyosarcoma, for these therapeutics.

*CHEK2* encodes the checkpoint kinase CHK2 involved in DNA damage repair and is implicated as a risk for cancer development [30]. Germline pathogenic variants of CHEK2 are associated with reduced overall survival in breast cancer patients [31]. Interestingly, we identified a pathogenic *CHEK2* variant in 1.4% of patient samples, which was the only DDR gene alteration significantly associated with prolonged overall survival. 

*ERCC2* encodes the Xeroderma Pigmentosum Complementation group D (XPD) protein, which is involved in the NER of damaged DNA [32]. Homozygous mutations in *ERCC2* are classically associated with the UV sensitivity conditions Xeroderma Pigmentosa and trichothiodystrophy, but have also been reported in approximately 11% of urothelial carcinomas and 2% of all solid tumors [33]. In a large study determining sarcoma risk, rare variant burden analysis identified germline *ERCC2* variants in 23 patients with various sarcoma types, suggesting that *ERCC2* may be a newly recognized sarcoma susceptibility gene [6]. Our findings of *ERCC2* mutations in 3% of epithelioid sarcoma and 6.45% of perivascular epithelioid cell tumors, the trend for increased HRD scores among *ERCC2*-mutated sarcomas, and the trend for shorter overall survival among patients with *ERCC2* mutation support the further investigation of the role of *ERCC2* in sarcoma pathogenesis and treatment.

Although DDR mutations are implicated in tumorigenesis, they can also be used as biomarkers of targeted therapy. Therapies targeting the DDR pathways include topoisomerase inhibitors, checkpoint inhibitors, platinum-based therapies [4], and more recently, PARP inhibitor (PARPi) therapy. Recent investigations have begun to evaluate PARPi therapy in sarcoma patients, particularly Ewing sarcoma and leiomyosarcoma [34,35,36]. Limited information on the genetic profiles and sensitivities of the different sarcoma subtypes has prevented the widespread use of individualized, targeted therapy in sarcomas overall [37].

HRD assays may be a useful tool in identifying sarcoma patients who may benefit from PARPi therapy. Our findings suggest that certain histologic subtypes with high rates of HRD (≥10% observed in pleomorphic sarcoma, leiomyosarcoma, myxofibrosarcoma, other uterine sarcoma, perivascular epithelioid cell tumor, uterine sarcoma/mesenchymal, sarcoma not otherwise specified, and inflammatory myofibroblastic tumor), may be good candidates for further investigation of PARPi therapy. Also of note, Ewing sarcoma, which is known to be driven by *EWSR1* fusions, has preclinical data supporting sensitivity to PARPi therapy [38], yet had a 0% HRD rate and 3.45% DDR pathway alteration rate, among the lowest of all groups; to date, clinical trials testing PARPi have resulted in mixed responses [35,39]. Results from a recent phase 2 clinical trial (NCT03880019) investigating the PARPi Olaparib with Temozolomide in leiomyosarcoma patients found that Olaparib with Temozolomide provided meaningful clinical benefit in patients with advanced, pretreated uterine leiomyosarcoma [36].

Mismatch repair deficiencies (dMMR) have been used as a biomarker to predict response to immune checkpoint inhibitors (ICIs), making the pathway a focus for recent studies [40,41,42]. dMMR is uncommon in sarcomas, with only 1% of samples previously reported to be dMMR among 353 bone and 539 soft tissue tumors, and another study reported 0.7% dMMR among angiosarcoma samples [8]. Consistent with these studies, MMR gene alterations were rare in our study, including *MSH3* (0.6%), *MSH6* (0.5%), *MSH2* (0.3%), *MLH1* (0.3%), and *PMS2* (0.3%).

Additionally, DDR deficiencies have been associated with upregulation of PD-L1 [5]. A recent comprehensive genomic and immune profiling study showed a high detection of immune cell infiltration in the tumor microenvironment in genomically complex dedifferentiated liposarcoma, leiomyosarcoma, undifferentiated pleomorphic sarcoma, and myxofibrosarcoma, which was also highly associated with clinical outcome [14]. Similarly, in our study, DDR pathway alterations were associated with increased frequency of PD-L1+ IHC overall (+7.3%) and among rhabdomyosarcoma (+39.1%), phyllodes tumor of the breast (+37%), undifferentiated uterine sarcoma (+27.9%), and sarcoma NOS (+9.3%) subtypes (all *p* < 0.05). Several recent studies have investigated the role of ICIs in sarcoma patients. [34,35,36,37,38,39]. Although a number of studies have found high response rates to ICI therapy, alveolar soft parts sarcoma are typically thought to have low TMB [43,44]. We found alveolar soft parts sarcoma to have one of the highest DDR pathway alteration frequencies among sarcoma subtypes (25%), which may provide an explanation for the ICI responses reported for patients with alveolar soft parts sarcoma in these trials, independent of high TMB. To date, there are no clinical trials investigating ICI response in sarcoma with inclusion based on dMMR/MSI-H or other predictive biomarkers. The evidence for dMMR/MSI-H status to predict response to ICI therapy, along with the ICI responses observed for alveolar soft parts sarcoma, makes this an attractive hypothesis for such a study.

A prior study found that the expression of PARP1, γH2AX, BRCA1, and BRCA2 was associated with shorter survival of patients with sarcoma [45]. Our study found a significant decrease in overall survival among all patients with a DDR mutation compared to DDR-WT. We did not observe age-specific differences in overall survival between DDR-mut and DDR-WT patients.

Limitations of our study include a lack of detailed clinical information (staging, treatment received), a lack of central pathologic review and classification, use of OncoTree classification with inclusion of certain benign histologies such as desmoid fibromatosis, lack of germline testing, and the use of insurance claim data to estimate patient survival. While *TP53* has been associated with the DDR pathway through the transcriptional activation of DDR genes, it is not considered a DDR gene by itself. Importantly, *TP53* influences several other pathways related to cell cycle regulation and stress, and therefore was excluded from our analysis; however, we acknowledge that it may be influencing some of the survival results. Further, we were unable to test associations between DDR alterations and clinical outcomes in specific sarcoma subtypes, and the enrichment of sarcoma subtypes with generally good or bad prognosis may impact outcomes independent of DDR mutation status.

## 5. Conclusions

In conclusion, we provide an in-depth study of somatic DDR pathway mutations and immune signatures in sarcoma. In many subtypes, DDR-mut tumors were found to have increased rates of PD-L1+, dMMR/MSI-high, and TMB-high biomarkers commonly used to identify patients that may benefit from immunotherapy. We note shorter overall survival in DDR-mut patients in the overall sarcoma cohort. These data offer new insights and highlight the need for additional studies for a better understanding of the DDR alteration in sarcoma to help identify further prognostic features and therapeutic options for these rare cancers.

## Figures and Tables

**Table 1:** Patient demographics by sarcoma subtype.

Sarcoma Subtype	Abbreviation	Sample Size	Sex Female % (N)	Age Median (Range)	Ped/AYA 0–39 Yrs % (N)
Overall	Overall	5309	56.6% (3007)	60 (0–90+)	14.1% (746)
Leiomyosarcoma	LMS	1005	80.3% (807)	60 (19–90+)	4.6% (46)
Gastrointestinal Stromal Tumor	GIST	872	48.3% (421)	64 (11–90+)	6.2% (54)
Sarcoma, NOS	SARCNOS	686	49.9% (342)	61 (0–90+)	15.9% (109)
Liposarcoma	LIPO	437	38.4% (168)	64 (14–90+)	6.9% (30)
Pleomorphic Sarcoma	PLSARC	278	42.1% (117)	68 (14–90+)	5.8% (16)
Angiosarcoma	ANGS	260	60.4% (157)	69 (6–90+)	7.3% (19)
Spindle Cell Sarcoma	SCSARC	150	49.3% (74)	62.5 (0–90+)	14.0% (21)
Rhabdomyosarcoma	RMS	130	49.2% (64)	38.5 (0–85)	50.0% (65)
Synovial Sarcoma	SYNS	119	46.2% (55)	46 (15–86)	33.6% (40)
Solitary Fibrous Tumor/Hemangiopericytoma	SFT	103	49.5% (51)	60 (0–86)	9.7% (10)
Chondrosarcoma	CHS	96	37.5% (36)	52 (17–84)	18.8% (18)
Osteosarcoma	OS	94	30.9% (29)	29 (6–78)	61.7% (58)
Malignant Peripheral Nerve Sheath Tumor	MPNST	91	36.3% (33)	40 (1–90+)	45.1% (41)
Chordoma	CHDM	88	42.0% (37)	61 (1–87)	14.8% (13)
High-Grade Endometrial Stromal Sarcoma	HGESS	82	100.0% (82)	58 (26–79)	9.8% (8)
Uterine Carcinosarcoma/Uterine Malignant Mixed Mullerian Tumor	UCS	75	100.0% (75)	66 (38–88)	1.3% (1)
Myxofibrosarcoma	MFS	68	45.6% (31)	64.5 (6–90+)	5.9% (4)
Ewing Sarcoma	ES	58	41.4% (24)	32 (4–86)	63.8% (37)
Desmoid/Aggressive Fibromatosis	DES	57	59.6% (34)	39 (1–82)	47.4% (27)
Undifferentiated Uterine Sarcoma	UUS	46	100.0% (46)	62 (30–83)	8.7% (4)
Epithelioid Hemangioendothelioma	EHAE	41	43.9% (18)	54 (12–76)	24.4% (10)
Fibrosarcoma	FIBS	41	34.1% (14)	67 (14–90+)	12.2% (5)
Uterine Sarcoma, Other	OUSARC	36	100.0% (36)	62.5 (19–89)	2.8% (1)
Low-Grade Endometrial Stromal Sarcoma	LGESS	35	100.0% (35)	54 (21–81)	14.3% (5)
Uterine Adenosarcoma	UAS	35	100.0% (35)	63 (36–87)	2.9% (1)
Hemangiopericytoma of the Central Nervous System	HPCCNS	33	51.5% (17)	49 (24–73)	30.3% (10)
Round Cell Sarcoma, NOS	RCSNOS	33	54.5% (18)	35 (5–84)	57.6% (19)
Epithelioid Sarcoma	EPIS	33	33.3% (11)	41 (17–80)	42.4% (14)
Perivascular Epithelioid Cell Tumor	PECOMA	33	84.8% (28)	55 (27–81)	3.0% (1)
Clear Cell Sarcoma	CCS	28	46.4% (13)	44.5 (19–84)	39.3% (11)
Myxoid Chondrosarcoma	MYCHS	27	22.2% (6)	56 (31–81)	3.7% (1)
Phyllodes Tumor of the Breast	PT	25	100.0% (25)	54 (19–88)	12.0% (3)
Inflammatory Myofibroblastic Tumor	IMT	23	60.9% (14)	57 (18–85)	26.1% (6)
Dedifferentiated Chondrosarcoma	DDCHS	22	63.6% (14)	60 (33–76)	4.5% (1)
Desmoplastic Small-Round-Cell Tumor	DSRCT	22	22.7% (5)	26.5 (14–77)	77.3% (17)
Uterine Sarcoma/Mesenchymal	USARC	22	100.0% (22)	60.5 (30–79)	9.1% (2)
Mesenchymal Chondrosarcoma	MCHS	13	46.2% (6)	36 (17–88)	61.5% (8)
Alveolar Soft Part Sarcoma	ASPS	12	58.3% (7)	22.5 (17–65)	83.3% (10)

## Data Availability

The datasets analyzed during the current study are not publicly available but are available from the corresponding author upon reasonable request.
